# Catalytic properties of Al_13_TM_4_ complex intermetallics: influence of the transition metal and the surface orientation on butadiene hydrogenation

**DOI:** 10.1080/14686996.2019.1608792

**Published:** 2019-05-29

**Authors:** Laurent Piccolo, Corentin Chatelier, Marie-Cécile De Weerd, Franck Morfin, Julian Ledieu, Vincent Fournée, Peter Gille, Emilie Gaudry

**Affiliations:** a Univ Lyon, Université Claude Bernard - Lyon 1, CNRS, IRCELYON, Villeurbanne, France; b Université de Lorraine, CNRS, IJL, Nancy, France; c Synchrotron SOLEIL, L’Orme des Merisiers, Saint-Aubin, France; d Department of Earth and Environmental Sciences, Crystallography Section, Ludwig-Maximilians-Universität München, München, Germany

**Keywords:** Complex intermetallic compounds, Al_13_Fe_4_, Al_13_Co_4_, Al_13_Ru_4_, heterogeneous catalysis, hydrogenation, butadiene, density functional theory, single-crystal surfaces, 60 New topics / Others, 106 Metallic materials, 205 Catalyst / Photocatalyst / Photosynthesis, 212 Surface and interfaces, 401 1st principle calculations

## Abstract

Complex intermetallic compounds such as transition metal (TM) aluminides are promising alternatives to expensive Pd-based catalysts, in particular for the semi-hydrogenation of alkynes or alkadienes. Here, we compare the gas-phase butadiene hydrogenation performances of *o-*Al_13_Co_4_(100), *m*-Al_13_Fe_4_(010) and *m*-Al_13_Ru_4_(010) surfaces, whose bulk terminated structural models exhibit similar cluster-like arrangements. Moreover, the effect of the surface orientation is assessed through a comparison between *o-*Al_13_Co_4_(100) and *o-*Al_13_Co_4_(010). As a result, the following room-temperature activity order is determined: Al_13_Co_4_(100) < Al_13_Co_4_(010) < Al_13_Ru_4_(010) < Al_13_Fe_4_(010). Moreover, Al_13_Co_4_(010) is found to be the most active surface at 110°C, and even more selective to butene (100%) than previously investigated Al_13_Fe_4_(010). DFT calculations show that the activity and selectivity results can be rationalized through the determination of butadiene and butene adsorption energies; in contrast, hydrogen adsorption energies do not scale with the catalytic activities. Moreover, the calculation of projected densities of states provides an insight into the Al_13_TM_4_ surface electronic structure. Isolating the TM active centers within the Al matrix induces a narrowing of the TM d-band, which leads to the high catalytic performances of Al_13_TM_4_ compounds.

## Graphical abstract

**Figure:**
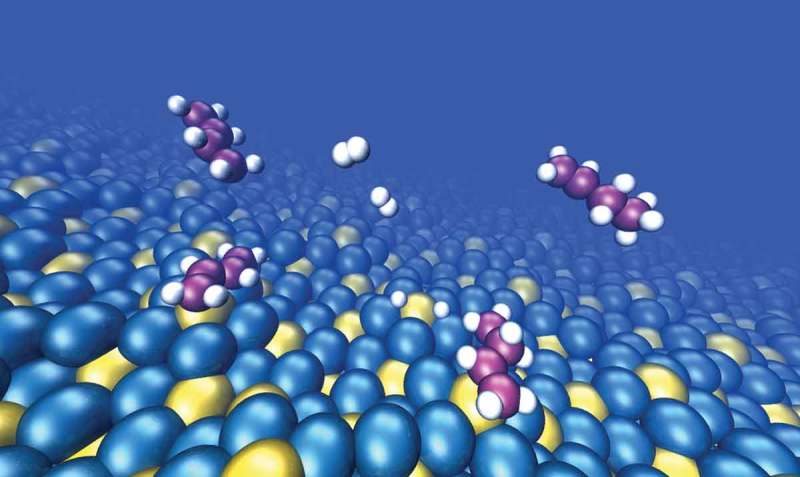

## 1. Introduction

The interest for intermetallic catalysts, i.e. compounds of at least two metallic and/or metalloid elements in the periodic table, with a defined crystal structure and stoichiometry, has considerably increased in recent years [1,2]. The potential of these compounds lies in their stability, which is ensured by strong metal-metal bonds with an iono-covalent character for several of them, and the possibility to tune the catalytic activity and selectivity based on electronic, geometric and ordering effects [2]. Several examples can be found among noble metal-containing intermetallics such as Pd-Ga [3–8], Pd-In [8], Pd-Zn [9], and Pt-Sn [10], which are able to selectively catalyze alkyne (one triple C-C bond, as in acetylene) or alkadiene (two double C-C bonds, as in butadiene) hydrogenation. These reactions are of great industrial importance for, e.g. the processing of petrochemical products for polymer synthesis.

Pd-based catalysts are rare and costly but actually used in industrial processes. The addition of a poorly active coinage metal (Ag, Au, Cu) is known to improve the catalyst selectivity to partially hydrogenated products [11,12–14]. For the semi-hydrogenation of acetylene, low-cost alternatives to reference Pd-Ag in the form of Ni-Zn alloys were discovered through a theoretical approach based on the density functional theory [15]. However, under reaction conditions, such substitutional alloys may suffer from adsorption-induced surface segregation, accompanied with a decrease in selectivity [12,16,17].

The recent identification of transition-metal (TM) aluminides such as Al_13_Fe_4_, Al_13_Co_4_, and Al_5_Co_2_ as efficient catalysts for the semi-hydrogenation of acetylene or butadiene is a major breakthrough in the quest for noble metal-free catalysts [18–25]. Indeed, the chemical bonding network in these polar intermetallics, which are also quasicrystal approximants, may prevent any surface segregation process, ensuring the structural stability of the catalyst. The interest for polar intermetallic compounds, i.e. compounds with lower valence electron concentrations than those of the Zintl phases, but close to those of the Hume–Rothery phases [26], is not limited to catalysis. They have recently received extensive attention as attractive materials for a wide range of applications (permanent ferromagnets, magnetocalorics, shape-memory alloys, superconductors, hydrogen storage materials, and thermoelectrics). Several studies have compared the physical properties of Al_13_TM_4_ compounds along the series (TM = Co, Fe, Ru, Rh), but no drastic differences were highlighted, in line with the similar vibrational and electronic properties [23]. These approximants to decagonal quasicrystalline phases present specific crystal structures, described as a stacking of atomic planes perpendicular to the pseudo 10-fold direction, each plane presenting pentagonal atomic arrangements [27]. This leads to an anisotropy in the magnetic properties, as well as in charge and heat transport properties [28–30].

The complex structure of such polar intermetallics, involving mixed iono-covalent bonding, may also be of interest for heterogeneous catalysis. Owing also to the site isolation concept [3] already invoked to explain the high performance of Al_13_Fe_4_ towards the semi-hydrogenation of acetylene [18], the catalytic properties of Al_13_TM_4_ compounds are thus expected to be quite different from those of their pure TM counterparts. The surfaces of these materials can expose Al pentagons centered by the TMs, which are believed to be the catalytically active centers [31]. Besides, Al_13_Fe_4_ has been recently proposed as a template for substituting single Pt atoms at Fe sites [32].

Here, we focus on the comparison of the catalytic properties of Al_13_TM_4_ compounds (with TM = Fe, Co, Ru) for the gas-phase hydrogenation of butadiene, which is an important petrochemical reaction for the purification of C4 cuts and the production of butenes before polymerization or alkylation [33–36]. This reaction is industrially performed on Pd-based catalysts, but it is also catalyzed by non-noble metals such as Fe, Co, and Ni, although with a much lower activity [37,38]. Our objective was to investigate the influence of the transition metal and the surface structure on the catalytic properties. Our rational approach combines experiments on oriented single-crystal surfaces and theoretical calculations based on ideal infinite surfaces within periodic boundary conditions. The combination of experimental measurements with theoretical calculations is crucial here. Indeed, surface-science studies achieved under ultra-high vacuum (UHV) have already highlighted that the pseudo 10-fold surface structures of these compounds differ: a surface reconstruction is observed for Al_13_Ru_4_(010) [39], but not for Al_13_Co_4_(100) and Al_13_Fe_4_(010) [40–42]. The surface structure and composition determined under UHV are also different between Al_13_Co_4_(100) and Al_13_Fe_4_(010). Nevertheless, catalytic properties are determined under gas pressure (typically a few millibars of reactants), which may imply different surface structures as compared to the UHV case. The consideration of a common surface model for all explored Al_13_TM_4_ pseudo 10-fold surfaces ensures a sound comparison between the catalysts, and may help to conclude about the influence of geometric or electronic factors.

The paper is organized as follows. Materials, then experimental and theoretical methods are described in Section 2. In Section 3, the catalytic measurements are detailed, highlighting the superior performances of Al_13_Fe_4_(010) and Al_13_Co_4_(010) with respect to Al_13_Ru_4_(010) and Al_13_Co_4_(100). A rationalization of the catalytic properties of the three Al_13_TM_4_ pseudo 10-fold surfaces is then proposed, based on electronic structure calculations including the shapes of the density of states and the adsorption energies. Our results are finally discussed in Section 4.

## 2. Methods

### 2.1. Materials

Centimeter-size Al_13_TM_4_ (TM = Co, Fe, Ru) single crystals were grown from an Al-rich solution using the Czochralski method [43] and polished with diamond paste down to 0.25 µm grain size. For the investigation of the catalytic properties, samples with surfaces oriented perpendicular to the pseudo-10-fold axis were cut from the ingot: *o*-Al_13_Co_4_(100),*m*-Al_13_Fe_4_(010) and *m*-Al_13_Ru_4_(010). An additional sample, *o*-Al_13_Co_4_(010), was used to investigate the influence of the surface orientation on the catalytic performances. In the following, when *o* or *m* prefixes are not mentioned, the crystal structures correspond to those of the above four samples.

### 2.2. Experimental methods

#### 2.2.1. Surface preparation

**Figure 1.**
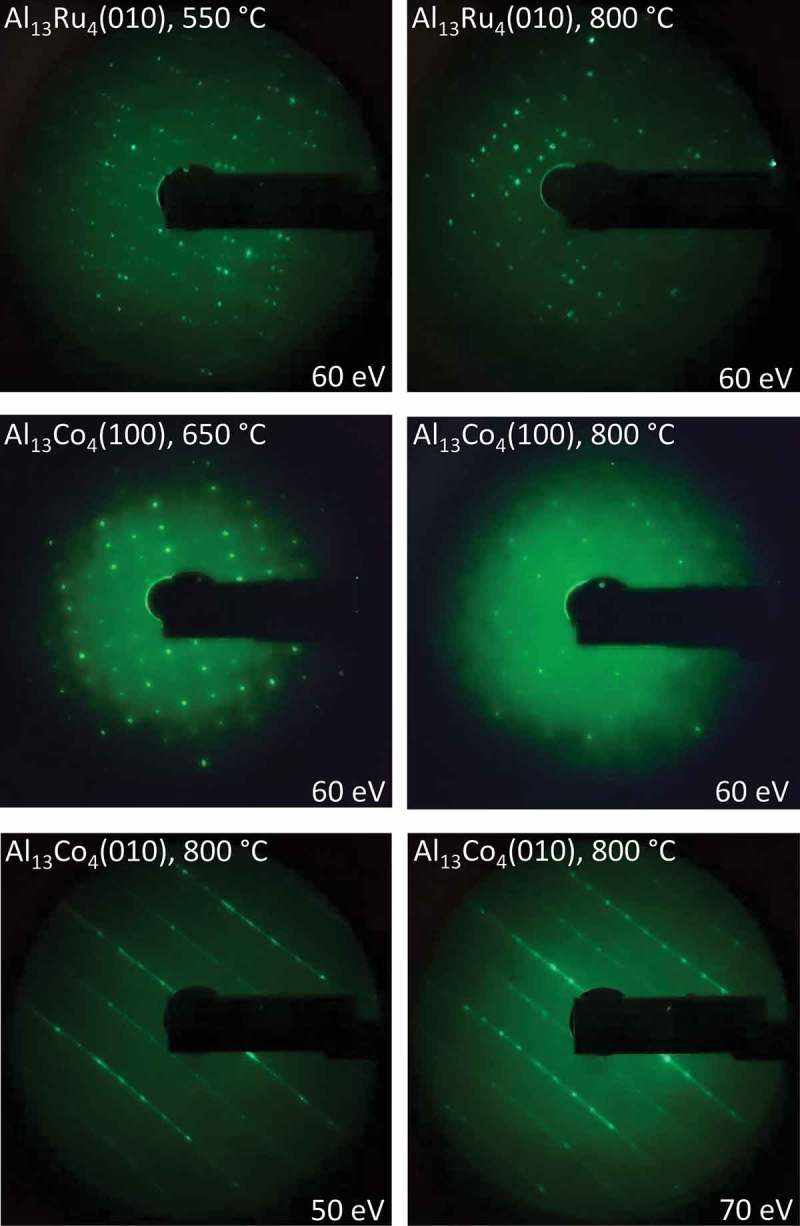
LEED patterns of clean annealed Al_13_Ru_4_(010), Al_13_Co_4_(100) and Al_13_Co_4_(010) surfaces. The approximate annealing temperature (°C) and the primary energy (eV) are indicated.

For each sample, the surface was cleaned by repeated cycles of Ar^+^ sputtering (2 kV) for 15 min and annealing for 20 min under UHV (base pressure ~5 × 10^–10^ Torr). The annealing temperatures were approximately 550–650°C and 800°C for Al_13_Co_4_(100), 750°C for Al_13_Fe_4_(010) [21], 550°C and 800°C for Al_13_Ru_4_(010), and 800°C for Al_13_Co_4_(010). Figure 1 and Figure S1 (Supplementary Information) show the low-energy electron diffraction (LEED) patterns and Auger electron spectra (AES), respectively, of the clean annealed surfaces (for Al_13_Fe_4_(010) the reader may consult Ref [21]). Figure S1 shows that no contaminants such as C or O were detected by AES on any surface following the sputtering-annealing procedure.

In the case of Al_13_Co_4_(100), the high-temperature (800°C) annealing [40] led to a more disordered surface than the low-temperature one (650°C), as shown by Figure 1. Conversely, the atomic order of Al_13_Ru_4_(010) increased with the annealing temperature (lower background intensity), although the atomic structure of the surface remained essentially unchanged (Figure 1), consistently with the work of Ledieu et al. [39]. For both Al_13_Co_4_(100) and Al_13_Ru_4_(010), the two annealing temperatures led to similar near-surface compositions, as probed by AES. In the following, the results for Al_13_Co_4_(100) and Al_13_Ru_4_(010) relate to low-temperature and high-temperature annealing, respectively, unless explicitly mentioned. For Al_13_Fe_4_(010) and Al_13_Co_4_(010), only high-temperature annealing was performed and led to sharp LEED patterns. In the case of Al_13_Co_4_(010), which had not been investigated so far, the main diffraction spots appear connected to each other through continuous and parallel lines (Figure 1). The corresponding surface structure is presently undetermined.

#### 2.2.2. Catalytic evaluation

The butadiene hydrogenation reaction was carried out in a dedicated static catalytic reaction cell (volume ca. 120 cm^3^) coupled to a surface preparation/analysis (AES/LEED) setup [44]. In the cell, the sample was investigated either at room temperature (RT, 24°C) or heated on the backside through a porthole using an infrared laser beam while the surface temperature was measured with an infrared pyrometer (surface emissivity set to 0.3). In a typical experiment, a mixture of butadiene (N26 purity), hydrogen (N55) and argon (N56) was prepared in a separate chamber before its injection into the reactor. All the gases were purchased from Air Liquide. The standard initial conditions in the reactor cell were 0.5 mbar butadiene, 5 mbar H_2_, and 0.5 mbar Ar (argon was used for internal calibration). At the end of the reaction run, the products were evacuated to secondary vacuum using a turbomolecular pump, and the reaction/evacuation cycle could be performed additional times.

During the reaction, the gases were continuously sampled through a leak valve and analyzed by a mass spectrometer (MS) evacuated by an oil diffusion pump capped with a liquid nitrogen trap (base pressure 2 × 10^–10^ Torr, analysis pressure 2 × 10^–8^ Torr). The MS intensities for m/z = 2, 40, 54, 56, and 58 were recorded for hydrogen, argon, butadiene (C_4_H_6_), butenes (C_4_H_8_), and butane (C_4_H_10_), respectively. In some cases, on-line gas chromatography (GC) was employed in addition to MS for determining the full distribution of 1,3-butadiene hydrogenation products, i.e. the three butene isomers (1-butene, *trans*-2-butene and *cis*-2-butene) and butane, using an automatic gas sampling device connected to an Agilent 6850 GC-FID [44]. With an Agilent HP-AL/KCl column (50 m × 0.53 × 15 µm) kept at 80°C, a chromatogram was recorded every 10 min.

### 2.3. Theoretical methods

#### 2.3.1. Bulk and surface models

**Figure 2.**
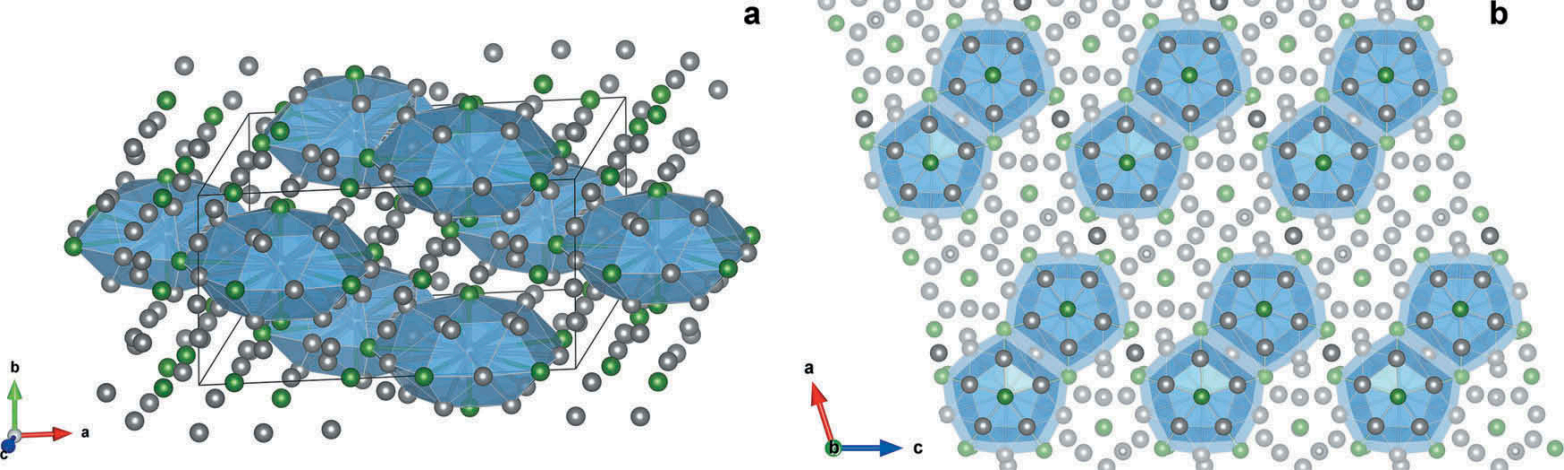
(a) Bulk structure of Al_13_Ru_4_ as a stacking of Henley-type clusters (represented in blue). The black cuboid represents the unit cell. (b) Top view  of the Al_13_Ru_4_(010) surface. Grey and green balls represent Al and Ru atoms, respectively. Shaded balls correspond to subsurface atoms. The crystal (a) and surface (b) directions are indicated by the unit vectors {**a, b, c**} and {**a, c**}, respectively.

The *o*-Al_13_Co_4_ compound crystallizes in the *Pmn*2_1_ space group [45], while *m*-Al_13_Fe_4_ and *m*-Al_13_Ru_4_ are monoclinic (C2/m space group) [46–48]. Their bulk structure (102 atoms per conventional cell,  see Figure 2in the case of Al_13_Ru_4_) consists in a stacking of two types of planes perpendicular to the pseudo-10-fold direction ([010] for *m*-Al_13_Fe_4_ and *m*-Al_13_Ru_4_, [100] for *o*-Al_13_Co_4_). Alternative descriptions involve polyhedral atomic arrangements, such as the ones proposed by Henley [49], based on geometric considerations.

The pseudo 10-fold surface models considered in this study are deduced from a cleavage keeping the cluster substructure intact at the surface (Figure 2). Each protruding surface TM atom is surrounded by a pentagonal arrangement of Al atoms, leading to the isolation of TM atoms at the surface. Under UHV, this surface model has been experimentally observed in the case of *m*-Al_13_Fe_4_(010), but not for *o*-Al_13_Co_4_(100) and *m*-Al_13_Ru_4_(010) [39,41,50]. However, some operating conditions may stabilize this model (Gaudry et al., in preparation), which has already been postulated to explain the catalytic activity of *o*-Al_13_Co_4_(100) towards the semi-hydrogenation of acetylene [31]. In addition, the consideration of the same surface model for all compounds allows comparing the influence of the electronic factor (nature of the TM) on the adsorption properties.

#### 2.3.2. Computational details

The theoretical ground state properties were deduced from calculations based on the density functional theory (DFT), using the plane wave Vienna *ab initio* simulation package (VASP) [51–54]. The interaction between the valence electrons and the ionic core was described using the projector-augmented wave (PAW) method [55,56] within the generalized gradient approximation (GGA-PBE) [57,58], considering the valences of the atoms to be 3s^2^3p^1^ (Al), 4s^1^3d^7^ (Fe), 4s^1^3d^8^ (Co),and 4p^6^5s^1^4d^7^ (Ru). The DFT-D3-BJ functional [59] was also used, since it is expected that the consideration of the van der Waals interactions influences the absolute values of the adsorption energies. The cut-off energy and the k-point grid were set to 450 eV and 5 × 9 × 1 (or equivalent), respectively, within the Brillouin zone. Spin polarization was not taken into account since it was shown to be unnecessary for such Al-rich complex intermetallic compounds [60]. The structures were plotted using the VESTA software [61].

Bulk structures were relaxed till the Hellmann-Feynman forces were as low as 0.02 eV/Å. The resulting theoretical bulk parameters are found in good agreement with the experimental ones. As shown by Table S1, the relative differences are smaller than 1% (resp. 2%) for Al_13_Co_4_ and Al_13_Ru_4_ (resp. Al_13_Fe_4_). Adsorption studies were performed using surfaces modeled with 7-layer-thick symmetric slabs separated by void (thickness ca. 12 Å). The positions of all the atoms in the slab were optimized, while the volume and the shape of the simulation cell were set. The adsorption energy for atomic hydrogen [*E_ads_* (H)], and 1,3-butadiene or 1-butene [*E_ads_* (but)] was calculated as the sum of the energies of the relaxed surface and the gas-phase molecule minus the energy of the adsorbate-surface system:

$$E_{ads}\left(H\right)=E\left(slab\right)\;+\;1\;/\;2E\left(H_{2}\right)-E\left(slab+H\right)$$

$$E_{ads}\left(but\right)=E\left(slab\right)\;+E\left(but\right)-E\left(slab+but\right)$$

where *E*(slab), *E*(H_2_), *E*(but), *E*(slab+H) and *E*(slab+but) are the energies of the systems {slab}, {dihydrogen molecule}, {unsaturated hydrocarbon molecule}, {slab + atomic hydrogen} and {slab + unsaturated hydrocarbon molecule}, respectively.

With this definition, stable adsorption on the considered site corresponds to a positive value of *E_ads_*.

Densities of states (DOS) of the d electrons *n_d_(ε)* were calculated for Al_13_TM_4_ compounds, as well as for the corresponding TMs (hcp Co, hcp Ru, bcc Fe). The d-band center is defined as:

(1) $$dbc=\frac{\int_{-\infty }^{E_{F}+1eV}\varepsilon n_{d}\left(\varepsilon \right)d\varepsilon }{\int_{-\infty }^{E_{F}+1eV}n_{d}\left(\varepsilon \right)d\varepsilon }$$

where ε represents the energy and *E_F_* the Fermi energy.

Projected DOS (pDOS) calculations were performed to investigate the molecule/surface interactions. The modifications of the molecular states following the adsorption process were evaluated from the comparison of the pDOS of the {molecule+surface} system, when the molecule is adsorbed at the surface and when the molecule is located far from the surface (distance larger than 10 Å).

## 3. Results

### 3.1. Experiments

**Figure 3.**
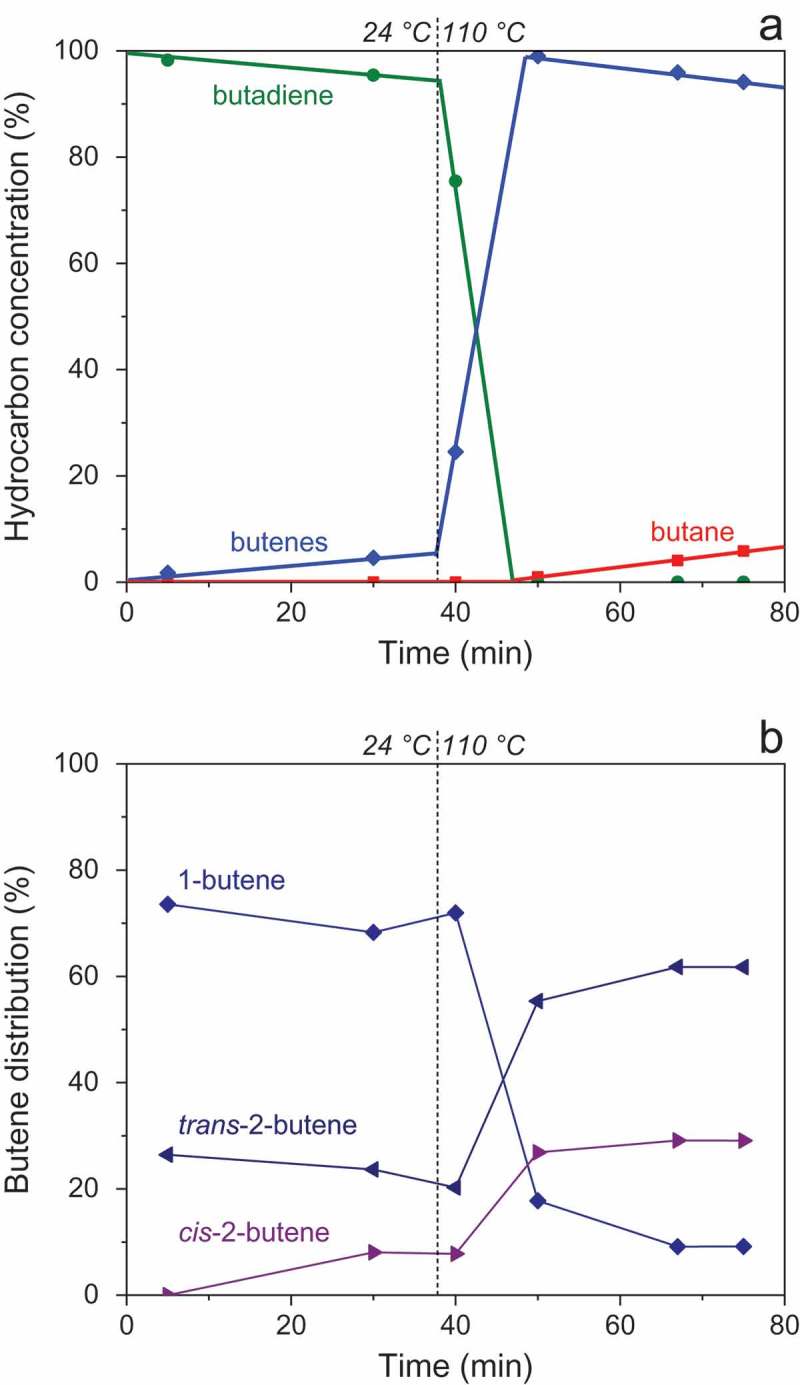
Hydrocarbon concentrations (a) and butene distribution (b) during butadiene hydrogenation over Al_13_Co_4_(010) at 24°C, then 110°C. The data points were obtained from GC analysis, whereas the lines in (a) were obtained from MS.

The clean annealed surfaces were exposed to a mixture of hydrogen (5 mbar) and butadiene (0.5 mbar) under batch conditions at RT and 110°C. In all cases except that of Al_13_Fe_4_, the reaction kinetics was slow at RT, i.e. only a small fraction of butadiene was converted after ca. 30 min, so that the tests at 110°C were generally performed during the same reaction run. Figure 3 shows the results of such an experiment for Al_13_Co_4_(010) (see also Fig. S2). Figures S3-S4 and S5-S6 show catalysis data for Al_13_Co_4_(100) and Al_13_Ru_4_(010), respectively. For detailed catalytic data concerning Al_13_Fe_4_(010), the reader may consult Ref [21].

Figure 3(a) shows that the selectivity of Al_13_Co_4_(010) to butenes is 100%, both at RT and 110°C, butane being formed only after complete butadiene conversion. The butene selectivity is also 100% for Al_13_Ru_4_(010) at any temperature (Fig. S5). For Al_13_Co_4_(100), it is 100% at RT at low conversion (Fig. S4(a)) and superior to 95% at 110°C (Fig. S3). The butene selectivity of Al_13_Fe_4_(010) is lower, but remains superior to 90% at any temperature. This lower selectivity correlates with the fact that Al_13_Fe_4_(010) is the only system, among the four Al_13_TM_4_ surfaces investigated, that exhibits a high activity for butene hydrogenation to butane (in the absence of butadiene). Butene hydrogenation over Al_13_Fe_4_(010) is even faster than butadiene hydrogenation in most conditions [21]. On the other surfaces, butene hydrogenation to butane is always slow (see Figure 3(a) for Al_13_Co_4_(010) and Fig. S5 for Al_13_Ru_4_(010), both at 110°C).

**Figure 4.**
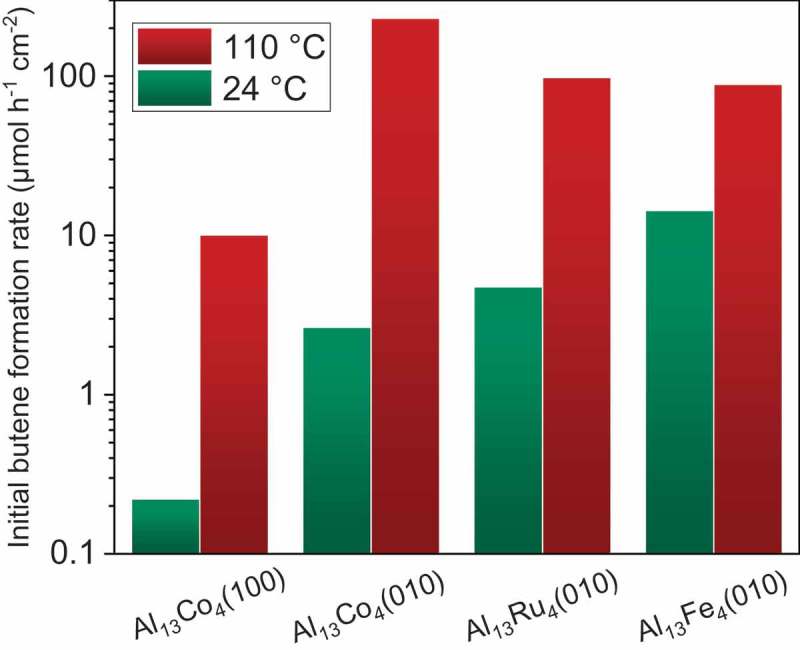
Comparison of initial butene-formation activities of Al_13_TM_4_ surfaces at RT and 110°C.

In terms of initial butene distribution at RT (1-butene:*trans*-2-butene:*cis*-2-butene = 74:26:0%, Figure 3(b)), Al_13_Co_4_(010) behaves more like Pd(100) (73:24:3%) than Al_13_Fe_4_(010) (71:13:16%). For the latter system, an unusually low *trans*/*cis* 2-butene ratio, close to unity, was indeed evidenced [21]. Conversely, for Al_13_Co_4_(010), *cis*-2-butene is not a primary product and its fraction remains lower than 10% throughout the reaction, as long as butadiene is present in the reactor, at both investigated temperatures. After butadiene conversion, the butenes distribution evolves towards a near-equilibrium value, 9:62:29%, through isomerization. As shown by Figure S4(b), the butenes distribution for Al_13_Co_4_(100) throughout the reaction is similar to that for Al_13_Co_4_(010) (64:27:9% for the former vs. 68:24:8% for the latter, at t = 30 min and RT), although the initial 1-butene fraction is lower for Al_13_Co_4_(100) (64% vs 74%). In contrast, the surface orientation has a dramatic effect on the catalytic activity, Al_13_Co_4_(010) being much more active than Al_13_Co_4_(100) both at RT and 110°C, as shown in Figure 4. Whereas in terms of activity at RT Al_13_Fe_4_(010) has no rival, Al_13_Co_4_(010) appears as the most active system at 110°C while keeping a butene selectivity of 100% at any temperature. The superstructure observed for Al_13_Co_4_(010) under UHV (Figure 1), if retained under reaction conditions, may play an important role in its good catalytic performance. Al_13_Ru_4_(010) exhibits an activity intermediate between those of Al_13_Fe_4_(010) and Al_13_Co_4_(010).

In the case of Al_13_Ru_4_(010), similar experiments were carried out at RT for the Ar ion-sputtered surface, the surface annealed at ca. 550°C, and the one annealed at ca. 800°C. As a result, the activity of the sputtered surface is lower than that of the 550°C-annealed surface, which is itself much lower than the activity of the (more structurally ordered) 800°C-annealed surface (Fig. S6). Similarly, a lower activity of the sputtered sample was previously measured for Al_13_Fe_4_(010) [20], and is also observed here in the case of Al_13_Co_4_(010) (Fig. S2). Moreover, AES shows that the sputtered Al_13_TM_4_ surfaces are richer in TM than the annealed surfaces (Fig. S1). These results indicate that the structural and chemical orders, and correlatively the active center isolation, are beneficial to the hydrogenation activity of the Al-TM systems.

Our previous work on Al_13_Fe_4_(010) had shown that this surface is easily contaminated by oxygen-containing reactant impurities, most probably H_2_O [20,21]. In the present work, when several subsequent reaction runs are performed on the same sample, the Co-containing surfaces show a loss of activity at any temperature (Figs. S2, S3), whereas Al_13_Ru_4_(010) deactivates at RT (Fig. S6) but not at 110°C (Fig. S5). Both Al_13_Co_4_(100) and Al_13_Co_4_(010) contain, after reaction, not only oxygen but also traces of carbon, while Al_13_Ru_4_(010) contains only a small amount of oxygen (Fig. S1). The higher resistance of Al_13_Ru_4_(010) is tentatively ascribed to the presence of the noble metal (Ru) instead of Co or Fe.

### 3.2. Calculations

On Al_13_Co_4_(100), a previous study suggested favorable sites for the adsorption of atomic hydrogen, acetylene (C_2_H_2_) and ethylene (C_2_H_4_) [31]. Based on these results, only one type of site per adsorbate has been considered here for the pseudo 10-fold surface of Al_13_TM_4_ compounds: a bridge Al-TM site for atomic hydrogen, di-π adsorption of 1,3-butadiene (C_4_H_6_) on neighboring Al and TM atoms, and π adsorption of 1-butene (C_4_H_8_) on a TM atom. The corresponding geometries and adsorption energies (*E_ads_*) are reported in Table 1.

**Table 1.** Adsorption configurations and energies (eV). The pictures were plotted using the Al_13_Ru_4_ model. Color code: Al = grey; Ru = green; C = red; H = black. Prefixes *o* and *m* relate to orthorhombic and monoclinic structures, respectively. PBE and D3 correspond to PBE and DFT-D3-BJ calculations, respectively.

		1,3-Butadiene	1-Butene	Hydrogen
*o*-Al_13_Co_4_(100)	PBE	1.06	0.62	0.18
	D3	1.59	1.13	0.24
*m*-Al_13_Fe_4_(010)	PBE	1.30	0.91	0.39
	D3	1.80	1.38	0.41
*m*-Al_13_Ru_4_(010)	PBE	1.16	0.77	0.39
	D3	1.67	1.24	0.49

**Figure 5.**
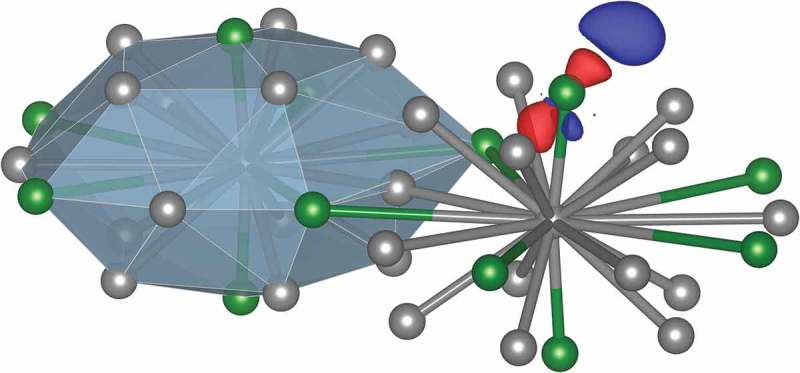
Charge deformation following atomic hydrogen adsorption, highlighting the TM-H interaction (isodensity 0.007 Å^−3^, plotted for H on Al_13_Ru_4_(010); the influence of the neighboring Al atom is not visible for this isodensity value). Charge accumulation and depletion are plotted in blue and red, respectively. The H atom is located inside the big blue distribution.

For atomic hydrogen adsorption, our result for Al_13_Co_4_(100) (*E_ads_^PBE^* = 0.18 eV) is in good agreement with the value reported in Ref [31] (*E_ads_^PBE^* = 0.21 eV), and shows that atomic hydrogen is much less strongly bonded to Al_13_Co_4_(100) than to Co(0001) (*E_ads_^PBE^* = 0.48 eV [62]). On Al_13_Fe_4_(010) and Al_13_Ru_4_(010), considering the same type of Al-TM site, atomic hydrogen is bonded slightly stronger (*E_ads_^PBE^* = 0.39 eV). Again, these adsorption energies are lower than those calculated for Fe(110) (*E_ads_^PBE^* = 0.54 eV) and Ru(0001) (*E_ads_^PBE^* = 0.55 eV) [63,64]. These trends are also valid within the DFT-D3-BJ approach. The charge density deformation, defined as the charge density difference between the adsorbate-surface system and the sum of the charge densities for the relaxed slab and the adsorbate, plotted in Figure 5 for H/Al_13_Ru_4_(010), suggests a metal-H bond with an iono-covalent character. This is also consistent with the fact that H adsorption energies do not scale with the Bader charges held by TM atoms of the TM-Al-TM molecular group in the bulk (−3.83*e* for Al_13_Fe_4_, −4.08*e* for Al_13_Co_4_, and −4.32*e* for Al_13_Ru_4_).

On the pseudo 10-fold surfaces, the adsorption energy of butadiene adsorbed in a di-π configuration increases from 1.06 eV (*o*-Al_13_Co_4_) to 1.16 eV (*m*-Al_13_Ru_4_) and 1.30 eV (*m*-Al_13_Fe_4_) within the PBE scheme (Table 1). The trends are similar when considering the van der Waals interactions, with *E_ads_*(butadiene) ranging from 1.59 eV (*o*-Al_13_Co_4_) to 1.67 eV (*m*-Al_13_Ru_4_) and 1.80 eV (*m*-Al_13_Fe_4_). The adsorption energies of 1-butene on the same surfaces are significantly lower, and the difference between *E_ads_*(butadiene) and *E_ads_*(butene) is almost constant (0.39–0.46 eV), regardless of the used functional.

Within the d-band model of Nørskov and coworkers, the d-band position and width were shown to be key parameters for rationalizing the adsorption properties of transition-metal surfaces [65,66]. This model was further developed to include the influence of the shape of the electron DOS [67]. To illustrate the role played by the TM atom isolation within the Al matrix, and in turn the electronic structure influence on the adsorption properties, we first compare the Al_13_Ru_4_(010) surface and the (low-energy) Ru(0001) surface, for which the adsorption energies of C_4_H_6_ are calculated to be 1.16 eV (Table 1) and 1.71 eV, respectively. On Ru(0001), only one adsorption site and configuration have been considered for C_4_H_6_, as shown in Figure S7.

**Figure 6.**
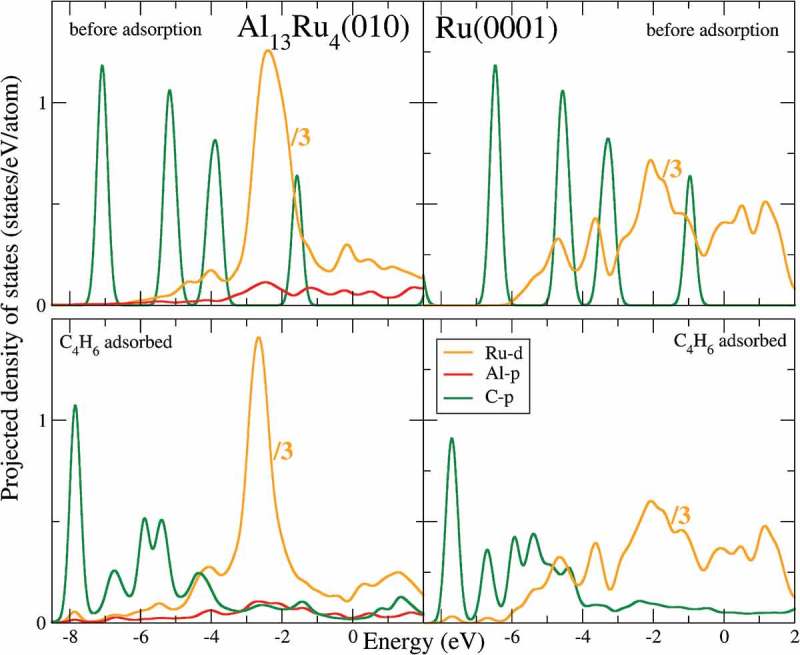
Projected density of states (pDOS): average C-p contribution of carbon atoms (butadiene, in green); Al-p (red) and Ru-d (orange) contributions of the closest Al and Ru atoms protruding at the surface and involved in the adsorption for Al_13_Ru_4_(010); Ru-d (orange) contribution of surface Ru atoms involved in the adsorption for Ru(0001). The Fermi energy was set to 0 eV.

The average contribution of the C-p orbital states in the butadiene molecule, as well as the Ru-d states involved in the adsorption, are represented in Figure 6. For Ru(0001), the contribution of Ru-d to the DOS is expanded on a much larger energy range (from the Fermi energy *E_F_* up to binding energies of 6–8 eV) than for Al_13_Ru_4_(010), for which it is mostly localized around 2–3 eV. For Ru(0001), the contribution of Ru-d to the DOS is rather uniform on a large energy range (up to a binding energy of 6 eV), while the Ru-d states are mostly localized around 2–3 eV for Al_13_Ru_4_(010). In both cases, the shapes and positions of the molecular states before adsorption (energies around −6.5 eV, −4.5 eV, −3.4 eV, and −1.0 eV) are modified upon adsorption, and new electronic states can be seen for E ≥ −8 eV, including states at the Fermi level arising from spd hybridization between the molecular and the surface states. In addition, a shift of the higher binding C-p state, located around −6.5 eV before adsorption and around −7.7 eV after adsorption, is visible for the Ru(0001) and Al_13_Ru_4_(010) surfaces, consistent with electron transfer from the substrate to butadiene.

**Figure 7.**
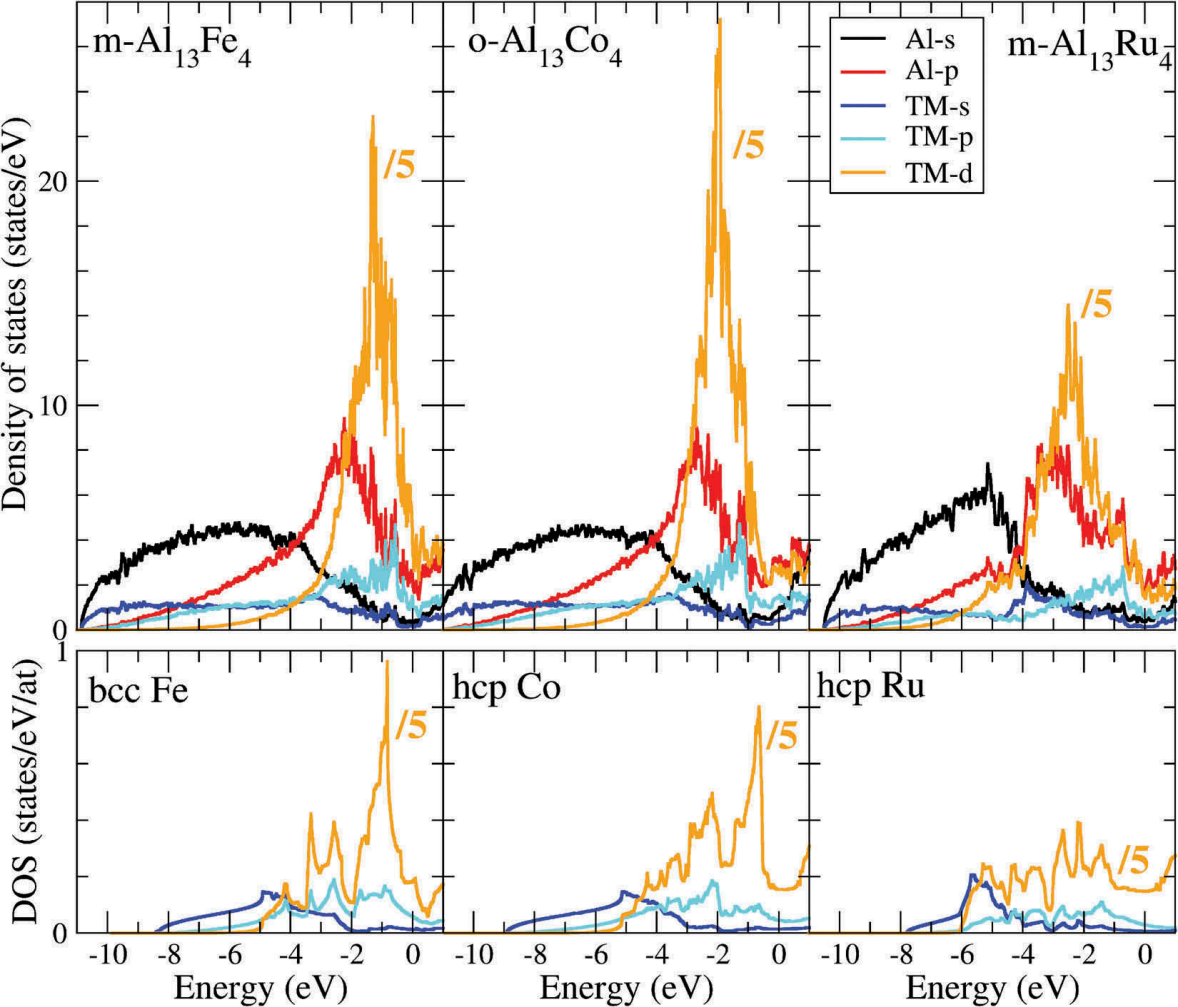
Contributions of s-, p-, and d-states to the density of states (DOS) for bulk Al_13_TM_4_ and TM. The Fermi energy corresponds to 0 eV.

To further look for a possible relationship between the d-band center position with respect to the Fermi energy (*ΔE_dbc_*) and the catalytic performances, the bulk electronic structures of Al_13_Ru_4_, Al_13_Fe_4_ and Al_13_Co_4_ are compared in Figure 7. The DOSs of Al_13_Fe_4_ and Al_13_Co_4_ are similar, with a free electron-like shape at low energy, and a strong maximum at higher energy caused by localized and weakly dispersive TM-d states. These DOSs considerably differ from those of the pure metals (bottom graphs in Figure 7), which exhibit a much more spread d-band, attributed to TM-TM hybridization. The latter is almost absent for Al_13_TM_4_ compounds, owing to the rather large nearest-neighbor TM-TM distance.

The stronger adsorption of butadiene on Al_13_Fe_4_(010) than on Al_13_Co_4_(100) (Table 1), classically attributed to a decreased filling of the adsorbate-metal antibonding states [68], is indeed in agreement with the lower-energy d-band center in the former case (*ΔE_dbc_* (Al_13_Fe_4_) = 1.41 eV, *ΔE_dbc_*(Al_13_Co_4_) = 1.97 eV). However, the previous scheme is not consistent with the results found for Al_13_Ru_4_(010) (*E_ads_* = 1.15 eV, *ΔE_dbc_* = 2.48 eV), indicating that other parameters, such as the d-band width and shape, likely play a role.

## 4. Discussion

The comparison of butadiene-hydrogenation activities of Al_13_TM_4_ surfaces (Figure 4) reveals a variety of behaviors, from the poor activity of Al_13_Co_4_(100) at any temperature to the high activities of Al_13_Fe_4_(010) at RT and Al_13_Co_4_(010) at 110°C; Al_13_Ru_4_(010) shows intermediate activity, but superior stability. This implies that (i) for the pseudo 10-fold surfaces, Fe is the most active center; (ii) depending on the surface orientation, Co acts as a highly [(010)] or poorly [(100)] active center. If the Al_13_Co_4_(100) surface actually present in catalytic conditions has the same structure as under UHV, i.e. a bulk-terminated Al-rich termination without protruding Co atoms, it is indeed expected to be poorly active. However, since on the one hand the exact surface structure of Al_13_Co_4_(010) under UHV is unknown, and on the other hand any of the systems may reconstruct under reaction conditions, choice has been made to compare the electronic structures of unreconstructed, cluster-terminated pseudo 10-fold surfaces through DFT calculations. This has allowed us to focus, for a given atomic structure, on the effect of the TM nature (Ru, Co, Fe) and isolation in an Al matrix on its surface electronic structure and corresponding bonding to the relevant adsorbates. As a result, site isolation is seen to considerably narrow the Ru d-band (Figure 6), which leads to an adsorption energy reduced by 1/3. From the comparison of the bulk electronic structures of TM and Al_13_TM_4_ systems (Figure 7), we have seen that TM isolation in the Al_13_TM_4_ structure proceeds similarly for the three TMs, i.e. by a narrowing of the d-band. Remarkably, the situation is here quite different from the one described in Ref [69], where the stronger adsorption on the Ag-Cu ‘single-atom alloy’ dilute in Cu, with respect to pure Cu, is attributed to the narrower d-band of the alloy.

Butadiene/butene adsorption energies (Table 1) follow the same order as hydrogenation activities: Al_13_Fe_4_ > Al_13_Ru_4_ > Al_13_Co_4_. Hence, in this (low) adsorption energy range (1.1–1.3 eV for butadiene within PBE), the hydrocarbon adsorption energy can be considered as a semi-quantitative descriptor of the hydrogenation activity. On the pure metals, the much higher adsorption energies of the hydrocarbons may be detrimental to hydrogenation rates, as suggested by the lower activities of the TM-rich sputtered surfaces (SI). Moreover, the higher adsorption energy of butadiene with respect to butene is consistent with a preference for butadiene adsorption and subsequent hydrogenation, i.e. to a high (near 100%) selectivity to butene [12,70]. In the sole case of Al_13_Fe_4_ butene appears sufficiently adsorbed to be (fastly) hydrogenated [21], whereas butene hydrogenation on the other Al_13_TM_4_ surfaces is very slow at any temperature (Section 3.1). The high butene-hydrogenation activity of Al_13_Fe_4_(010) is consistent with a selectivity to butene slightly lower than 100% in most butadiene-hydrogenation conditions.

In addition, the butenes distribution, and more specifically the *trans*/*cis* 2-butene ratio, is different between Al_13_Co_4_ (close to 3 for both orientations, and even much higher at the beginning of the reaction) and Al_13_Fe_4_ one (close to 1). Moyes et al. studied a number of TMs for butadiene hydrogenation and suggested that the electronic state of surface metal atoms would be the dominant factor driving the products distribution [38]. A high *trans*/*cis* 2-butene ratio was explained by the stabilization of π-allylic half-hydrogenated states which favored a selective pathway (as previously discussed for Al_13_Fe_4_(010) [21]) whereas πσ-adsorbed states favored *anti*/*syn* interconversion of the half-hydrogenated species and a more dispersed products distribution. Intermediate adsorbed species are not considered in the present work but a possible difference in the reaction pathways is consistent with the observed disparity in the apparent activation energy of the reaction over Al_13_TM_4_ compounds, i.e. Al_13_Co_4_(010) is the least active surface at RT but the most active one at 110°C (Figure 4).

As far as hydrogen adsorption is concerned, the adsorption energies for the Al_13_TM_4_ surfaces do not scale with the butadiene-hydrogenation activities (Table 1). However, the very low adsorption energy of H on Al_13_Co_4_(100) (E_ads_ < 0.2 eV) may contribute to the low hydrogenation activity of this surface.

In the future, a more quantitative theoretical description of the catalytic properties will have to include the effects of adsorbate coverage, thermodynamic conditions (pressure, temperature) and kinetics (reaction pathways including activation barriers).

## 5. Conclusions

On the basis of catalytic tests and DFT calculations, this work has allowed us to evidence trends in the electronic structures, adsorption properties and catalytic performances of the Al_13_TM_4_ intermetallic series. TM site isolation in the complex Al_13_TM_4_ structure induces a narrowing of the d-band and a decrease of the butadiene adsorption energy, leading to an enhanced hydrogenation activity. While Al_13_Fe_4_(010) is the most active model catalyst at room temperature, Al_13_Co_4_(010) is revealed as the most active one at higher temperature (110°C), while remaining 100% selective to butenes unlike Al_13_Fe_4_(010). In-depth investigations of Al_13_Co_4_(010) are needed to determine its structure and enable comparative DFT calculations. Moreover, a comprehensive understanding of the catalytic properties of these intermetallic surfaces requires *in situ* characterization (e.g. surface X-ray diffraction) to identify the actual working surface under gas-pressure conditions.
