# Supplementary material for: Catalytic properties of Al13TM4 complex intermetallics: influence of the transition metal and the surface orientation on butadiene hydrogenation
Source: Sci Technol Adv Mater. 2019 May 29;20(1):557–67. doi: 10.1080/14686996.2019.1608792 (PMC6586146; doi:10.1080/14686996.2019.1608792)
Supplement: Supplemental Material [file TSTA_A_1608792_SM2844.pdf]

Catalytic properties of $Al_{13}TM_4$ complex intermetallics: influence of the transition metal and the surface orientation on butadiene hydrogenation

Laurent Piccolo,[1*] Corentin Chatelier,[2,3] Marie-Cécile de Weerd,[2] Julian Ledieu,[2] Vincent Fournée,[2] Peter Gille,[4] Emilie Gaudry[2*]

[1] *Univ Lyon, Université Claude Bernard - Lyon 1, CNRS, IRCELYON - UMR 5256, 2 Avenue Albert Einstein, F-69626 VILLEURBANNE CEDEX, France*

[2] *Université de Lorraine, CNRS, IJL, F-54000 Nancy, France*

[3] *Synchrotron SOLEIL, L'Orme des Merisiers, Saint-Aubin – BP 48, F-91192 GIF-sur-YVETTE CEDEX, France*

[4] *Department of Earth and Environmental Sciences, Crystallography Section, Ludwig-Maximilians-Universität München, Theresienstr. 41, D-80333 München, Germany*

SUPPLEMENTARY INFORMATION

Table S1. Structural parameters of $Al_{13}TM_4$ compounds, obtained from theoretical and experimental studies. Prefixes o and m relate to orthorhombic and monoclinic structures, respectively. PBE and D3 correspond to PBE and DFT-D3-BJ calculations, respectively.

		a **(Å)**	***b*** **(Å)**	***c*** **(Å)**	β **(°)**
***o*-$Al_{13}Co_4$**	PBE [1]	8.20	12.40	14.42	
	D3	8.09	12.25	14.28	
	Exp. [2]	8.158	12.342	14.452	
***m*-$Al_{13}Fe_4$**	PBE [1]	15.43	8.02	12.43	107.69
	D3	15.27	7.92	12.29	107.69
	Exp. [3,4]	15.492	8.078	12.471	107.69
***m*-$Al_{13}Ru_4$**	PBE [1]	15.94	8.30	12.82	107.76
	D3	15.79	8.12	12.65	107.75
	Exp. [5,6]	15.86	8.19	12.74	107.77

[1] Scheid P, Chatelier C, Ledieu J, Fournée V and Gaudry É 2019 Bonding network and stability of clusters: the case study of Al13TM4 pseudo-tenfold surfaces *Acta Cryst A* **75** 314–24

[2] Grin J, Burkhardt U, Ellner M and Peters K 1994 Crystal structure of orthorhombic Co_4Al_{13} *J. Alloys Compd.* **206** 243–7

[3] Grin J, Burkhardt U, Ellner M and Peters K 1994 Refinement of the Fe_4Al_{13} structure and its relationship to the quasihomological homeotypical structures *Z. Kristallogr.* **209** 479–87

[4] Sugiyama K, Obata T and Hiraga K 2012 The Crystalline Structure of O-AlFePd *Mater. Trans.* **53** 1357–62

[5] Edshammar L-E, Nyberg B and Vänngård T 1965 The Crystal Structure of Ru_4Al_{13} *Acta Chem. Scand.* **19** 2124–30

[6] Murao R, Genba M, Sugiyama K and Sun W 2011 The Structure of an Al-Ni-Ru Monoclinic Phase $Al_{13}(Ru,Ni)_4$ *Mater. Trans.* **52** 1344–8

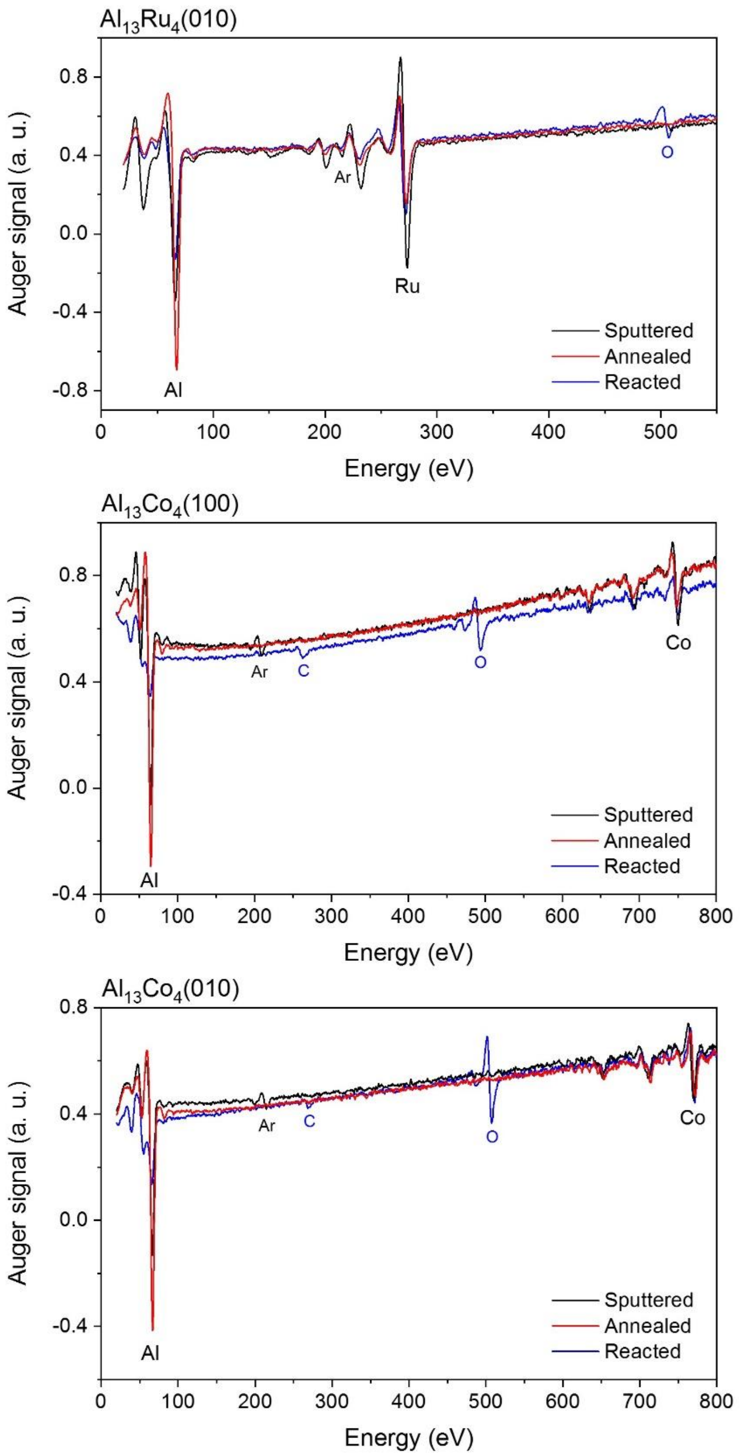

Figure S1. Auger spectra of clean Ar^+-sputtered (black) and annealed (red) $Al_{13}Ru_4(010)$, $Al_{13}Co_4(100)$ and $Al_{13}Co_4(010)$ surfaces. The spectra in blue were recorded after reaction at room temperature.

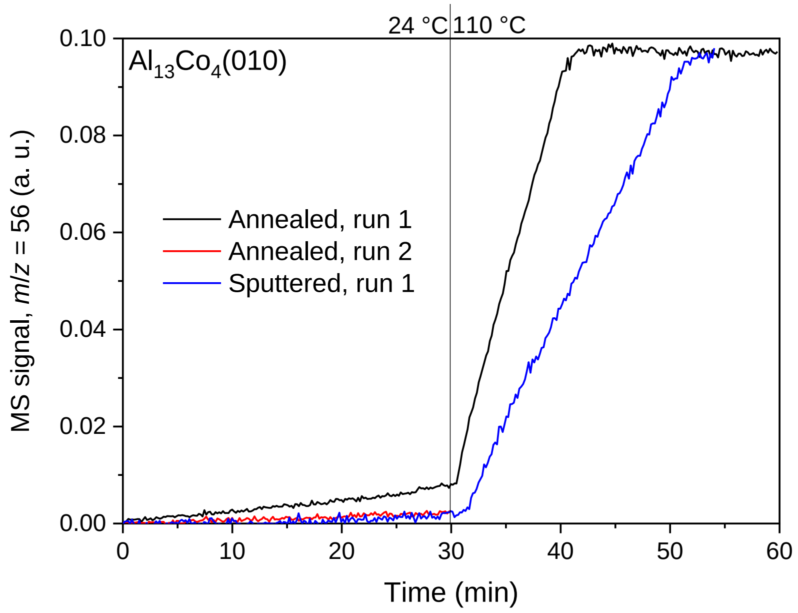

Figure S2. Evolution of the butene MS signal (with background signal subtracted) during butadiene hydrogenation at 24 °C, then 110 °C, on the clean annealed (black) and sputtered (blue) $Al_{13}Co_4$(010) surfaces. The signal in red corresponds to the second run on the annealed surface.

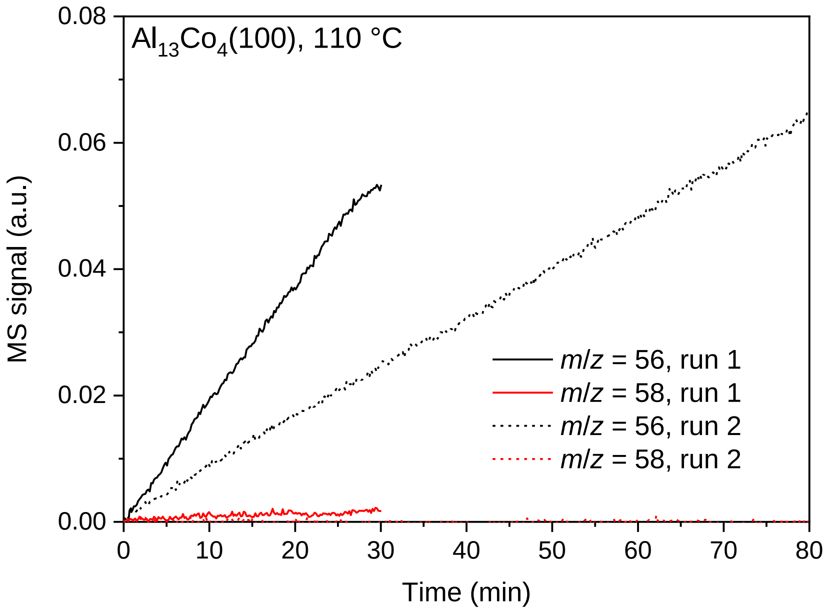

Figure S3. Evolution of the butene (black) and butane (red) MS signals (with background signal subtracted) during butadiene hydrogenation at 110 °C on $Al_{13}Co_4$(100). The dotted lines correspond to the second run on the same system.

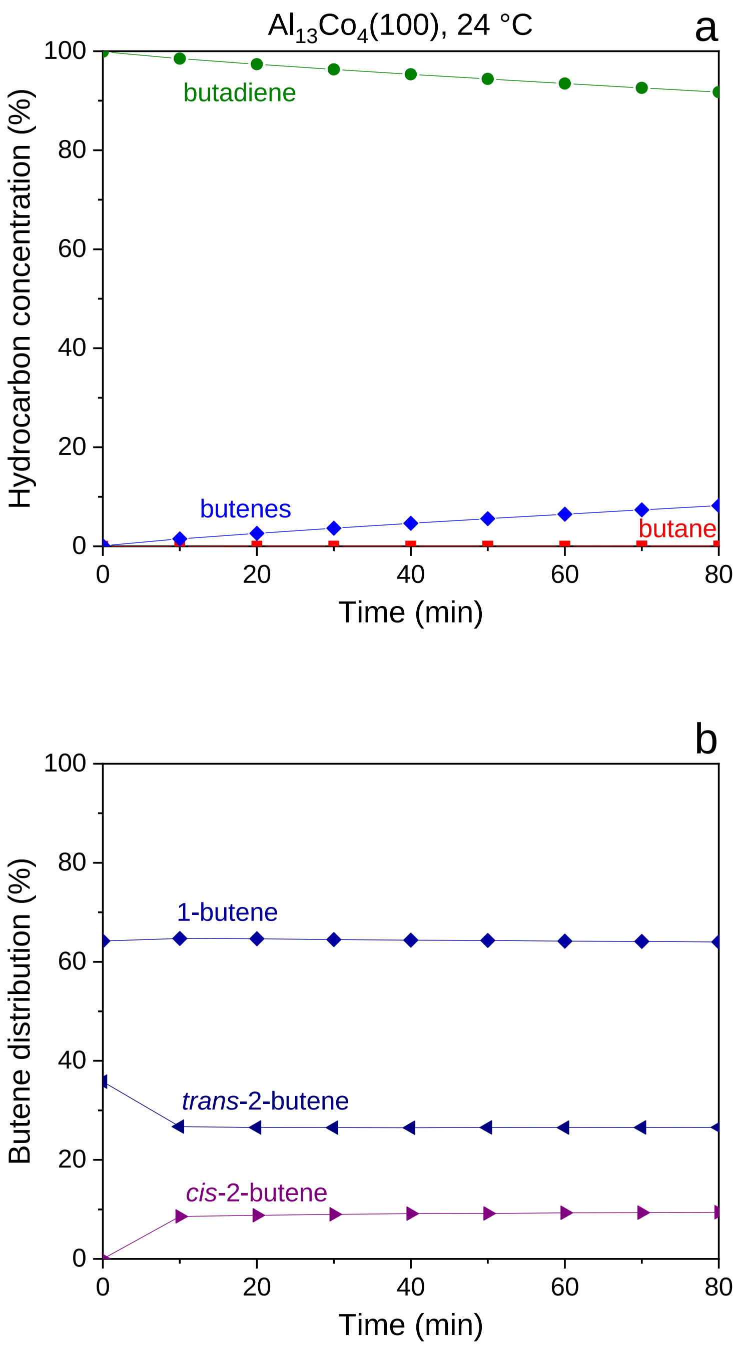

Figure S4. Hydrocarbon concentrations (a) and butene distribution (b) during butadiene hydrogenation over $Al_{13}Co_4$(100) at 24 °C. The data points were obtained from GC analysis.

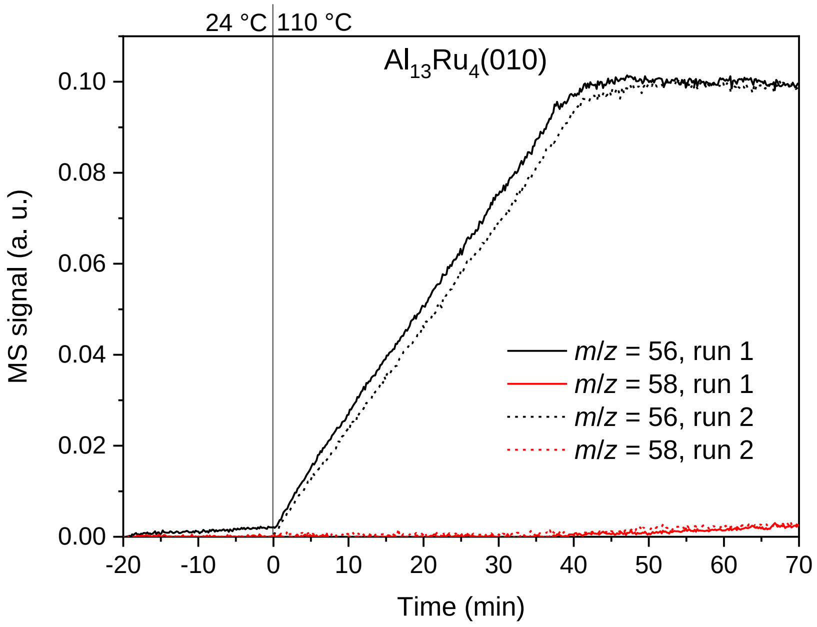

Figure S5. Evolution of the butene (black) and butane (red) MS signals (with background signal subtracted) during butadiene hydrogenation at 24 °C, then 110 °C, on the clean 800 °C-annealed $Al_{13}Ru_4$(010) surface. The dotted lines correspond to the second run at 110 °C on the same system.

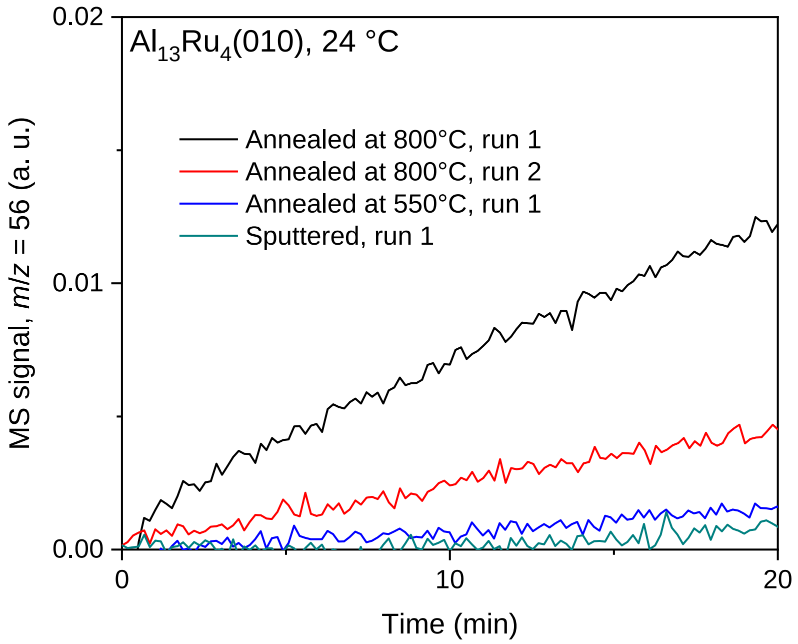

Figure S6. Evolution of the butene MS signal (with background signal subtracted) during butadiene hydrogenation at 24 °C on the clean 800 °C-annealed (black), 550 °C-annealed (blue), and sputtered (green) surfaces. The signal in red corresponds to the second run on the 800 °C-annealed surface.

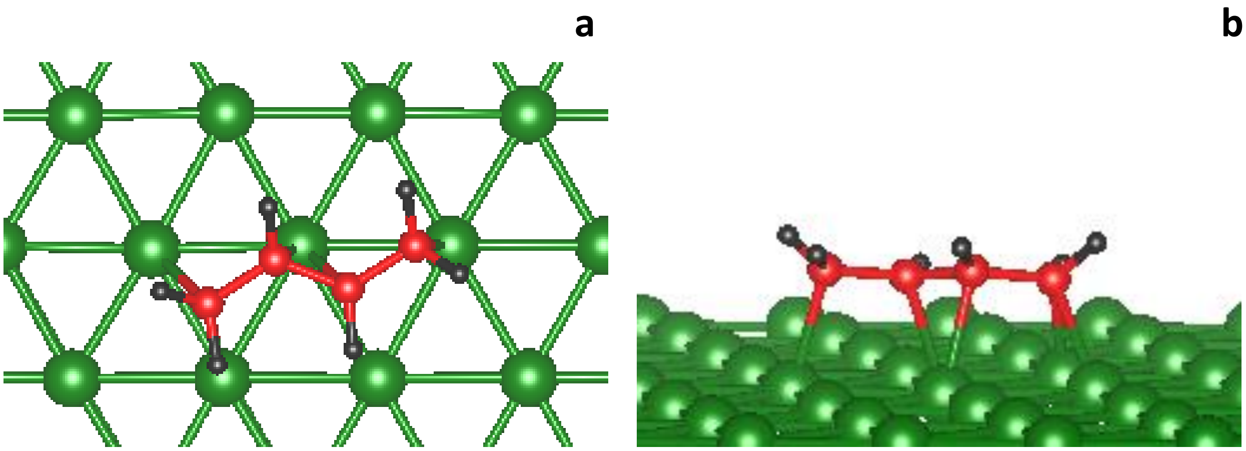

Figure S7. DFT model of butadiene adsorption on Ru(0001).
